# Microbiologically Pure Cotton Fabrics Treated with Tetrabutylammonium OXONE as Mild Disinfection Agent

**DOI:** 10.3390/ma15217749

**Published:** 2022-11-03

**Authors:** Bogdan Bujnicki, Przemyslaw Sowinski, Tomasz Makowski, Dorota Krasowska, Patrycja Pokora-Sobczak, Inna Shkyliuk, Józef Drabowicz, Ewa Piorkowska

**Affiliations:** 1 Centre of Molecular and Macromolecular Studies Polish Academy of Sciences, Sienkiewicza 112, 90-363 Lodz, Poland; 2 The Bio-Med-Chem Doctoral School of the University of Lodz and Lodz Institutes of the Polish Academy of Sciences, University of Lodz, Banacha 12/16, 90-237 Lodz, Poland; 3 Institute of Chemistry, Jan Dlugosz University in Czestochowa, Armii Krajowej 13/15, 42-200 Czestochowa, Poland

**Keywords:** cotton fabric, functionalization, tetrabutylammonium groups, microbiological purity, mechanical properties

## Abstract

The microbiological purity of textiles plays a pivotal role in the use of textiles, especially in hospitals and other medical facilities. Microbiological purity of cotton fabric was achieved by a new disinfection method using tetrabutyloammonium OXONE (TBA-OXONE) before washing. As a result of the disinfection, the cotton fabric became microbiologically pure, despite the markedly decreased washing time with respect to the widely used standard procedure. Shortening of the washing time allowed for significant energy savings. In addition, the effect of the number of disinfection and washing cycles on the tensile properties and tearing force of the fabric was examined. After 120 disinfection and washing cycles the mechanical properties of cotton fabric were only slightly worsened.

## 1. Introduction

Antibacterial finishing of textiles is increasingly important as it reduces the possibility of infections. It plays a pivotal role in the use of textiles in hospitals and other medical facilities. Widely used antibacterial agents embrace inorganic and organic compounds, some of them of natural origin. They include Ag and Cu nanoparticles [1,2,3,4], chitosan [4,5,6], quaternary ammonium compounds [7,8], *N*-halamine [9,10], triclosan [4], and others.

Antimicrobial agents can be deposited on textile substrates by a range of methods, including padding, coating, and spraying. The agents inhibit microbial growth by different mechanisms, which embrace: preventing cell reproduction, reacting and destructing the cell membranes, blocking enzymes, and poisoning the cells [11].

The textiles used in medical facilities, like hospitals, have to be frequently washed and disinfected. The applied disinfectants should be easy to use and safe. Moreover, they should not adversely affect the performance of the fabric and its appearance.

The disinfectants that are applied during industrial washing mainly belong to two groups of formulations. The first group is characterized by a low pH value and contains oxidizing agents, most often a mixture of acetic acid (CH_3_COOH), peracetic acid (CH_3_COOOH, pH = 1), and hydrogen peroxide (H_2_O_2_) is used (e.g., Ozonite, Septonite, Clax Personril 4KL5, Power Classic), also with a small amount (<0.8%) of sulfuric (VI) acid (H_2_SO_4_) (e.g., Peracid Asepsis) [12]. The second group of formulations includes sodium and potassium salts or their mixtures with inorganic peroxides and organic additives, e.g., a mixture of sodium carbonate (Na_2_CO_3_), sodium perborate (Na_2_B_2_O_4_(OH)_4_), polyoxyethylene glycol alcohols C_12_-C_16_, hydrate of sodium metasilicate (Na_2_SiO_3_x9H_2_O), alkanesulfonic acid (Alk-SO_3_H), and benzenesulfonic acid (C_6_H_5_SO_3_H) (e.g., Clovin II Seption) [13]. The requirements for disinfectants of cotton textiles include a neutral pH value (≈7). The disinfectants should be also nontoxic, and volatile, and not deteriorate the colors and mechanical performance of the textiles. The above limitations eliminate organic and inorganic acids as well as peracids. The mixture of 1 mol of the stable potassium salt of monopersulfuric (VI) acid (KHSO_5_), 0.5 mol of potassium salt of hydrosulfuric (VI) acid (KHSO_4_) and 0.5 mol of potassium sulfate (VI) (K_2_SO_4_), is an excellent example of an oxidant as strong as Caro acid [14]. Ammonium salts of the above-mentioned peracids with the addition of H_2_SO_4_ in dichloromethane easily oxidize hydroxyimines to ketones.

The potassium monopersulfate mixture 2KHSO_5_xKHSO_4_xK_2_SO_4_, known as OXONE, is commercially available. In the presence of ketones OXONE decomposes, but with acetone very reactive and unstable dioxirane structure (DMDO) is formed easily, as shown further, and oxidizes alkenes with excellent yields in dichloromethane-water biphasic system [15]. Oxidation occurs in the presence of labile functional groups sensitive to acids (pH < 7) or basis (pH > 7). The oxidation power of OXONE/acetone system is comparable with that of *m*-chloroperbenzoic acid (*m*-Cl-C_6_H_4_-C(O)OOH) [16,17]. Conversion of polycyclic aromatic hydrocarbons with OXONE to their oxides is possible e.g., 9–10 phenanthrene oxide is formed with 60% yield [18]. Epoxides are formed during oxidation with stronger oxidants [19,20] but the use of only OXONE in excess (1.5–2 equiv.) increases the yield to 95% [21,22]. Catalysts are not required during oxidation with OXONE for shortening of the reaction time, and the excess oxidant easily converts heterocyclic nitrogen compounds to their *N*-oxides [23]. Ylides are more sensitive in the reaction with OXONE than the compounds with carbon-carbon double bonds C=C. Even trisubstituted double bonds are not oxidized in bilayer benzene-water system [24], however, five and six-membered cycloalkenones lead to lactones [25] in dichloromethane and in the presence of wet aluminum oxide Al_2_O_3_ (yield up 80%). Moreover, organic quaternary ammonium salts are known as very strong antibacterial agents [26,27].

The potassium monopersulfate mixture 2KHSO_5_xKHSO_4_xK_2_SO_4_, known as OXONE, is commercially available. In the presence of ketones OXONE decomposes, but with acetone very reactive and unstable dioxirane structure (DMDO) is formed easily, as shown further, and oxidizes alkenes with excellent yields in dichloromethane-water biphasic system [15]. Oxidation occurs in the presence of labile functional groups sensitive to acids (pH < 7) or basis (pH > 7). The oxidation power of OXONE/acetone system is comparable with that of *m*-chloroperbenzoic acid (*m*-Cl-C_6_H_4_-C(O)OOH) [16,17]. Conversion of polycyclic aromatic hydrocarbons with OXONE to their oxides is possible e.g., 9–10 phenanthrene oxide is formed with 60% yield [18]. Epoxides are formed during oxidation with stronger oxidants [19,20] but the use of only OXONE in excess (1.5–2 equiv.) increases the yield to 95% [21,22]. Catalysts are not required during oxidation with OXONE for shortening of the reaction time, and the excess oxidant easily converts heterocyclic nitrogen compounds to their *N*-oxides [23]. Ylides are more sensitive in the reaction with OXONE than the compounds with carbon-carbon double bonds C=C. Even trisubstituted double bonds are not oxidized in bilayer benzene-water system [24], however, five and six-membered cycloalkenones lead to lactones [25] in dichloromethane and in the presence of wet aluminum oxide Al_2_O_3_ (yield up 80%). Moreover, organic quaternary ammonium salts are known as very strong antibacterial agents [26,27].

In the study, we have demonstrated that tetrabutylammonium salt of OXONE (TBA-OXONE) is a very efficient disinfection agent for cotton fabric. This compound was not used previously for such a purpose. Disinfection with TBA-OXONE ensured the microbiological purity of the cotton fabric despite the shortening of the industrial washing cycle with respect to the widely used standard procedure. Moreover, the use of TBA-OXONE did not markedly deteriorate the mechanical properties of the fabric.

## 2. Materials and Methods

The widely applied standard procedure of cotton fabric washing is The Christeyns Laundry Procedure [28] in water with Power Classic, which comprises two prewashing steps at 45 °C, four washing steps at 65 °C, and three rinsing steps, for 5 min each, then pressing for 3 min to decrease the amount of water, and drying at 120–140 °C, followed by ironing at 180 °C for 30–60 s. The procedure is additionally explained in Supplementary Information (SI). The washing machine Tunel Vega P9/40 is used for this purpose. Such a long washing with the above-mentioned agent causing very low pH (about 1) of the bath is time and energy consuming and leads to deterioration of the mechanical properties of cotton fabrics.

To shorten the washing, disinfection agents were prepared and used. The microbial purity of disinfected fabric samples was tested, which enabled to select the effective agent. The cotton fabric samples were then disinfected and washed, and the effect of the number of disinfection and washing cycles on their microbiological purity was tested as well as on the mechanical properties.

### 2.1. Materials

White plain weave cotton fabric, commercially available, with a surface density of 200 g/m^2^, warp and weft density 264/10 cm and 308/10 cm, respectively, was used in the study. All reagents and solvents used were purchased from Sigma-Aldrich: potassium monopersulfate mixture 2KHSO_5_xKHSO_4_xK_2_SO_4_ known as OXONE (purity 98%), tetra*n*-butylammonium hydrosulfate (nBu_4_NHSO_4_) (purity 98%), dichloromethane anhydrous (CH_2_Cl_2_) (purity 99.8%) and acetone laboratory reagent (purity 99.5%).

The following disinfection agents were prepared: I—0.8% solution of OXONE in water, II, III—0.2% and 0.4% solutions of triple salt of OXONE (TBA-OXONE) (*n*Bu_4_N^+^)_5_x2HSO_5_-xHSO_4_-xSO_4_^−2^ in dichloromethane, respectively. TBA-OXONE was synthesized in the reaction between OXONE and *n*Bu_4_NHSO_4_ in a dichloromethane solution according to the described procedure [29,30,31,32]. The details of the synthesis of TBA-OXONE are given in SI.

### 2.2. Treatment of Fabric

The fabrics were sprayed with 15 mL/m^2^ of the disinfection agents and dried in air. Then, they were sprayed with acetone, prewashed, washed, rinsed, dried, pressed, and ironed similarly to the standard procedure described above. However, each of the prewashing, washing, and rinsing steps was shortened from 5 min to 3.5 min, which amounts to approx. 31 min in total.

### 2.3. Testing of Fabric

After the treatment with disinfection agents, the microbiological purity of the fabric samples was tested to verify the effectiveness of the sterilization process. Swabs taken from the fabric surfaces, using a phosphate buffer, were placed in test tubes filled with tryptic soya agar. Next, the agar was incubated at 36 °C ± 2 °C for 24–48 h ± 2 h, and the clarity of the agar in the test tubes was examined.

To examine the effect of the disinfection process on the mechanical properties of the fabric, the tensile test and the tearing test were carried out at room conditions. The shape of the specimens and their location in the fabric samples conformed to PN-EN ISO 13934-1:2013 and PN-EN ISO 13937-2:2002 standards, respectively. Instron 5582 machine was used for the purpose. The fabrics disinfected and washed 30 (P30), 60 (P60), 90 (P90), and 120 (P120) times, and the untreated fabric (P0), were examined. For the tensile tests rectangular specimens 50 mm wide, with a length exceeding 200 mm, were cut out, with their longer sides parallel either to the warp or to the weft directions. The specimens were gripped 200 mm apart, whereas a drawing rate was 20 mm/min, i.e., 10%/min. Values of force at break and strain at break were determined from the recorded force–strain dependencies. At least five specimens of each type were drawn for each fabric sample to calculate the average values of the mechanical parameters. For the tearing test, the trouser-shaped rectangular samples, with the same sizes as for the tensile tests were cut out. They were cut parallel to the longer side to form 25 mm wide trouser legs, the slit ending at 100 mm from the shorter specimen side. Both legs were gripped at the same distance from the slit end, and drawn at 100 mm/min. According to PN-EN ISO 13937-2:2002 standard, each force–strain plot was divided into four equal parts, beginning with the first peak of force and ending with the last peak. In each case, the force values of all force peaks, for which the increase and decrease of the force was at least 10%, in the second, third and fourth intervals were determined, and the average value of force was calculated. At least five specimens of each type were tested for each fabric sample and the values of tearing force calculated for each specimen were averaged.

Moreover, the untreated fabric (P0) and the fabric disinfected and washed 120 times were examined using a scanning electron microscope (SEM) JEOL 6010LA (Tokyo, Japan), at an accelerating voltage of 10 kV. Before the examination, the fabric samples were vacuum sputtered with a 10 nm gold layer using Quorum EMS150R ES from Quorum Technologies (Laughton, UK).

## 3. Results and Discussion

**Figure 1:**
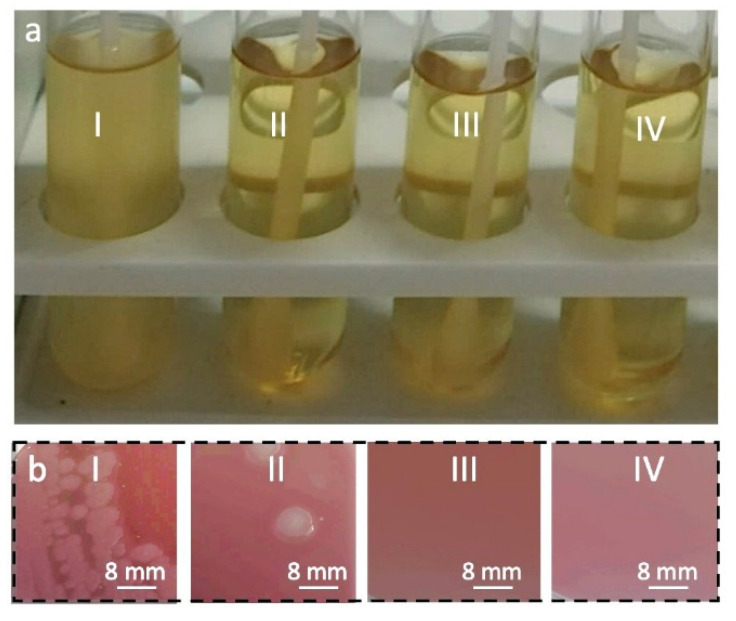
Analysis of microbiological purity of cotton fabric samples: (**a**) test tubes with agar after incubation, (**b**) photographs of the agar from the test tubes after incubation. Fabric sample not disinfected (**I**), fabric samples disinfected with 0.8% of OXONE (**II**); 0.2% TBA-OXONE (**III**); IV 0.4% TBA-OXONE (**IV**).

Figure 1 shows the results of the microbiological purity examination of the fabric samples. The turbidity of the agar in the test tubes is evidence of bacterial growth. The agar became turbid in the test tube with the swab taken from the fabric, which was not disinfected. On the contrary, the agar in the other three test tubes remained transparent. However, a more detailed examination of the agar in the tube with the swab taken from the sample disinfected with OXONE showed the presence of some bacterial colonization. Only the swabs taken from the samples disinfected with TBA-OXONE did not infect the agar, which remained clear after 48 h of incubation. It is worth noting that OXONE at a concentration of 0.8% did not show satisfactory antibacterial activity, as seen in Figure 1, whereas TBA-OXONE was efficient at a concentration of twice (0.4%) and four times (0.2%) lower.

There was the possibility that the number of disinfection and washing cycles can influence the disinfection efficiency, and after many cycles microorganisms would accumulate on the fabric surface. Hence, we repeated the disinfection and washing cycles 15, 30, 60, 80, 90, and 120 times. No microorganisms were detected on all fabric samples disinfected with TBA-OXONE regardless of the number of cycles.

**Figure 2:**
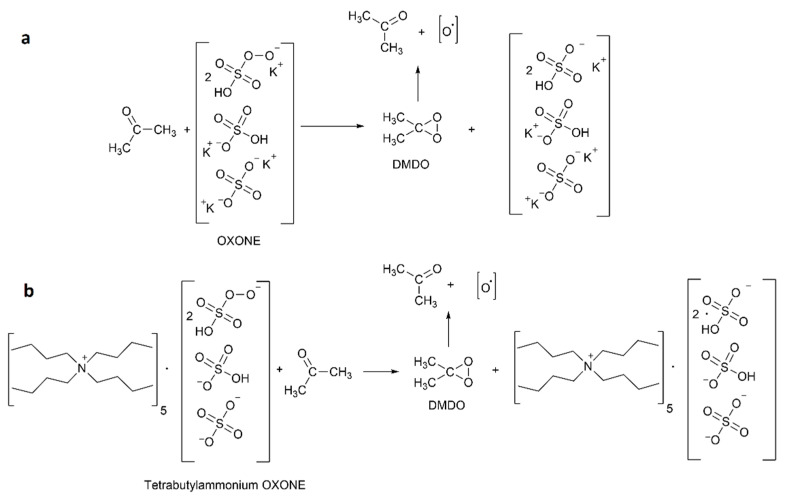
The comparison of the mechanisms of activity of two disinfecting agents: (**a**) OXONE, and (**b**) TBA-OXONE.

The mechanism of activity of OXONE and TBA-OXONE as disinfection agents is shown in Figure 2. In the first case (Figure 2a), the active element is the oxygen radical resulting from the decomposition of dimethyloxirane formed in situ in the reaction between OXONEs and acetone. In the second one (Figure 2b), in addition to oxygens, a strong oxidant antibacterial ammonium group is also present. For the disinfecting activity, a combination of antibacterial and oxidative properties is beneficial. During the reactions, dimethyloxirane (DMDO) was formed [33,34]. The low concentration (below 1%) of in situ generated DMDO is very important because the pure agent is explosive.

**Figure 3:**
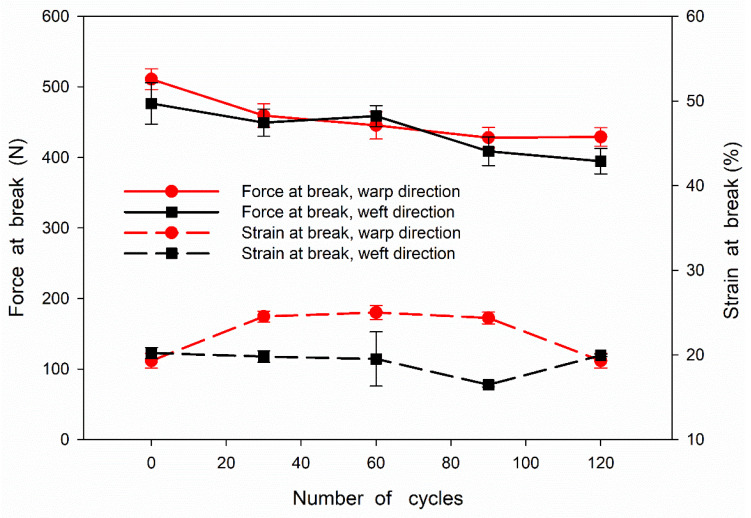
Dependencies of force and strain at break on number of disinfection and washing cycles of cotton fabric.

**Figure 4:**
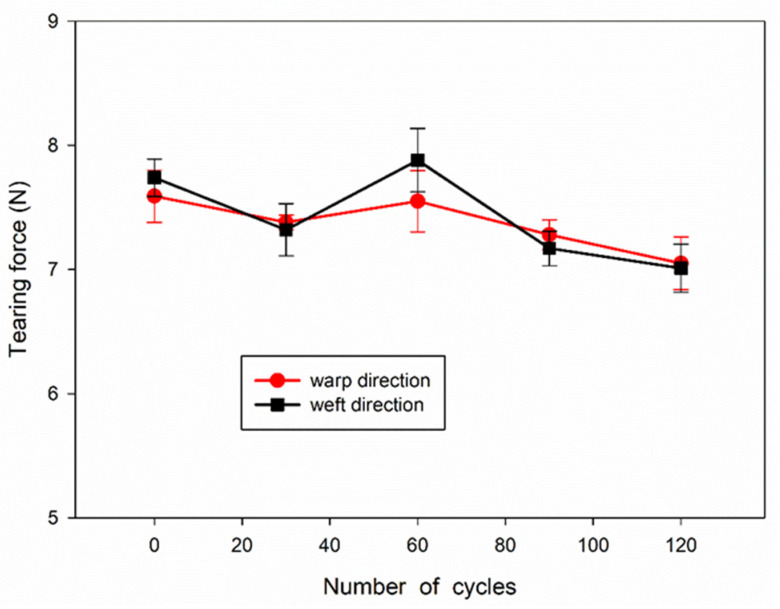
Dependence of tearing force on number of disinfection and washing cycles of cotton fabric.

0.2% concentration of TBA-OXONE in dichloromethane was sufficient. Thus, the mechanical properties of the fabrics treated with this agent and washed were tested. The results obtained for the fabrics disinfected and washed 30 (P30), 60 (P60), 90 (P90), and 120 (P120) times, and the untreated fabric (P0), are shown in Figure 3 and Figure 4.

The dependence of force at break and strain at break on the number of disinfection and washing cycles of the tested fabric samples is shown in Figure 3. It can be seen that the force at break during drawing in the warp and weft direction decreased from 510 to 430 N and from 480 to 390 N, respectively, with increasing number of cycles. Thus, the decrease of the force at break did not exceed 15 and 20%, respectively. In turn, the strain at break during drawing in the warp and weft direction varied within the ranges of 19–25% and 20–16%, respectively, without a clear dependence on the number of cycles. The dependence of tearing force on the number of disinfection and washing cycles is shown in Figure 4. The tearing force showed a tendency to decrease slightly with increasing number of cycles, from 7.7–7.6 N to 7.0 N, that is by 10% after 120 cycles. It can be concluded that the disinfection and washing cycles did not significantly deteriorate the mechanical properties of the fabric. It is worth mentioning that a slight decrease in the strength of cotton fabric solely due to the antibacterial treatment was also observed by others [35].

**Figure 5:**
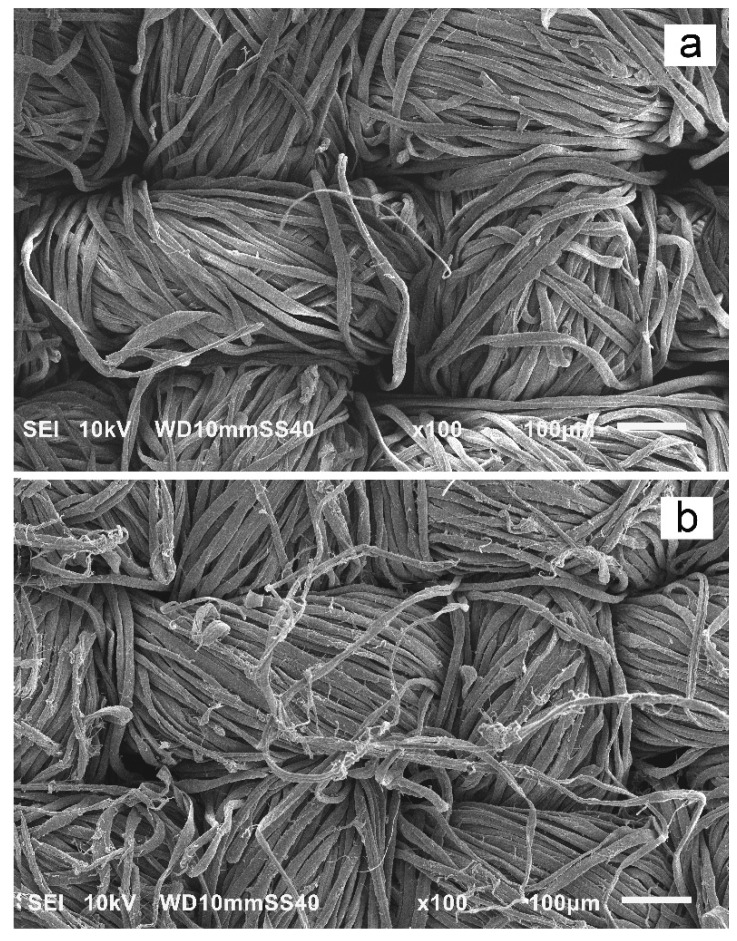
SEM micrographs of cotton fabric samples: (**a**) untreated (P0), and (**b**) disinfected and washed 120 times (P120).

SEM micrographs of the untreated fabric (P0) and the fabric disinfected and washed 120 times (P120) are compared in Figure 5. It is seen that 120 disinfection and washing cycles resulted in the damage of some fibers on the thread surfaces, which correlates with the slight decrease of mechanical performance.

## 4. Conclusions

OXONE and TBA-OXONE were tested as disinfection agents for cotton fabric. Fabric samples were sprayed with the disinfection agents and next with acetone, before washing. The washing cycle was shortened with respect to the standard washing procedure. Evidence of microbial colonization was found on the fabric samples not disinfected or disinfected with an 0.8% solution of OXONE in water. However, the use of 0.2% and 0.4% solutions of TBA-OXONE in dichloromethane as disinfection agents permitted to impart of antibacterial activity to the fabric and ensured its microbiological purity. The microbiological purity remained after disinfection with of 0.2% solution of TBA-OXONE in dichloromethane and industrial washing. This result was achieved despite the shortening of the washing cycle with respect to the widely used standard washing procedure. The shortening of the cycle by approx. 30 min allowed to save washing time and energy. Moreover, the mechanical properties of the fabric, the force and strain at break during drawing, and the tearing force were not significantly decreased even after 120 disinfection and washing cycles.

## Data Availability

Original data may be provided by the corresponding author in justified cases.

## Supplementary Materials

The following supporting information can be downloaded at: https://www.mdpi.com/article/10.3390/ma15217749/s1. Standard washing procedure of cotton fabric, Synthesis of TBA-OXONE.
