# Supplementary material for: Microbiologically Pure Cotton Fabrics Treated with Tetrabutylammonium OXONE as Mild Disinfection Agent
Source: Materials (Basel). 2022 Nov 3;15(21):7749. doi: 10.3390/ma15217749 (PMC9654703; doi:10.3390/ma15217749)
Supplement: Supplementary file 1 [file materials-15-07749-s001.zip › materials-1959962-supplementary.pdf]

Supplementary Information

Microbiologically Pure Cotton Fabrics Treated with Tetrabutylammonium OXONE as Mild Disinfection Agent

Bogdan Bujnicki [1,*], Przemyslaw Sowinski [1,*], Tomasz Makowski [1], Dorota Krasowska [1], Patrycja Pokora-Sobczak [1], Inna Shkyliuk [1,2], Józef Drabowicz [1,3,*] and Ewa Piorkowska [1]

[1] Centre of Molecular and Macromolecular Studies Polish Academy of Sciences, Sienkiewicza 112, 90-363 Lodz, Poland
[2] The Bio-Med-Chem Doctoral School of the University of Lodz and Lodz Institutes of the Polish Academy of Sciences, University of Lodz, Banacha 12/16, 90-237 Lodz, Poland
[3] Institute of Chemistry, Jan Dlugosz University in Czestochowa, Armii Krajowej 13/15, 42-200 Czestochowa, Poland
* Correspondence: bogbujni@cbmm.lodz.pl (B.B.); przem_so@cbmm.lodz.pl (P.S.); draj@cbmm.lodz.pl (J.D.); Tel.: +48-42-6803234 (B.B.); +48-42-6803316 (P.S.); +48-42-6803234 (J.D.)

Standard washing procedure of cotton fabric:

1. two prewashing steps (in consecutive chambers) at 45 °C for 5 min each
2. four main washing steps at 65 °C for 5 min each
3. three rinsing steps for 5 min each
4. pressing for 3 min to decrease the amount of water
5. drying at 120 – 140 °C
6. ironing at 180 °C for 30 – 60 s.

During the prewashing and washing the weight ratio of cotton fabric to water was 1:4 whereas during rinsing it was 1: 6.

Synthesis of TBA-OXONE

OXONE (1 equiv.) and varying amounts of nBu_4NHSO_4 were used to extract TBA-OXONE into CH_2Cl_2. By utilizing one equivalent of nBu_4NHSO_4 in a single extraction, 88% of the oxidative equivalence is obtained in soluble form. Additionally, it was not necessary to mix the solids for a longer time, because the simple mixing of both solids in water followed by immediate extraction provided reproducible results. The success of this procedure is attributed to the fact that

$KHSO_5$ dissolves preferentially over $KHSO_4$ and K_2SO_4, and that nBu_4NHSO_5 is more soluble in dichloromethane than nBu_4NHSO_4.

$$\text{n-Bu}_4\text{NHSO}_4 + \underbrace{2\text{KHSO}_5 + \text{KHSO}_4 + \text{K}_2\text{SO}_4}_{\text{OXONE}} \rightleftharpoons \underbrace{\text{n-Bu}_4\text{NHSO}_5}_{\substack{\text{more soluble in} \\ \text{CH}_2\text{Cl}_2}} + (\text{n-Bu}_4\text{N})_2\text{SO}_4$$
